# Dr. James Farquhar Hibberd

**Published:** 1903-10

**Authors:** 


Dr. James Farquhar Hibberd, of Richmond, Ind., died at his home in that city, September 8, 1903, aged 86 years. He was born at Monrovia, Md., and received his academic education at Alexandria, Va. He attended medical lectures at Yale University and received his degree at the College of Physicians and Surgeons, New York, in 1849. He practised his profession for a few years in California, removing to Richmond in 1856. He was professor of physiology and general pathology in the Ohio Medical College, Cincinnati, during the sessions of 1860 and 1861.

Dr. Hibberd was one of the founders of the Ohio State Medical Society, one of the chief organisers of the Indiana State

Medical Society, and served a term as the first vice-president of the American Medical Association in 1865; later its president in 1894. He was a member of many other local and national medical societies, in most of which he held official positions. He served for a time in the union army during the Civil War, was in the Ohio legislature for three terms, and from 1875 to 1877 was mayor of Richmond.

